# Tenecteplase in Pulmonary Embolism Patients: A Meta-Analysis and Systematic Review

**DOI:** 10.3389/fmed.2022.860565

**Published:** 2022-03-31

**Authors:** Zhu Zhang, Linfeng Xi, Shuai Zhang, Yunxia Zhang, Guohui Fan, Xincao Tao, Qian Gao, Wanmu Xie, Peiran Yang, Zhenguo Zhai, Chen Wang

**Affiliations:** ^1^Department of Pulmonary and Critical Care Medicine, China-Japan Friendship Hospital, Beijing, China; ^2^National Center for Respiratory Medicine, Beijing, China; ^3^Institute of Respiratory Medicine, Chinese Academy of Medical Sciences, Beijing, China; ^4^National Clinical Research Center for Respiratory Diseases, Beijing, China; ^5^Pulmonary and Critical Care Medicine, Capital Medical University, Beijing, China; ^6^Institute of Basic Medical Sciences, Chinese Academy of Medical Sciences, Peking Union Medical College, Beijing, China; ^7^Peking Union Medical College, Chinese Academy of Medical Sciences, Beijing, China; ^8^Department of Respiratory Medicine, Capital Medical University, Beijing, China

**Keywords:** tenecteplase, thrombolysis, meta-analysis, pulmonary embolism, efficacy and safety

## Abstract

**Objective:**

To assess the efficacy and safety of tenecteplase in patients with pulmonary embolism (PE).

**Methods:**

We completed the literature search on May 31, 2021 using PubMed, EMBASE and the Web of Science. Analyses were conducted according to PE risk stratification, study design and duration of follow-up. The pooled risk ratios (RRs) and its 95% confident intervals (CIs) for death and major bleeding were calculated using a random-effect model.

**Results:**

A total of six studies, with four randomized controlled trials (RCTs) and two cohort studies, were included in this study out of the 160 studies retrieved. For patients with high-risk PE, tenecteplase increased 30-day survival rate (16% vs 6%; P = 0.005) and did not increase the incidence of bleeding (6% vs 5%; P = 0.73). For patients with intermediate-risk PE, four RCTs suggested that tenecteplase reduced right ventricular insufficiency at 24h early in the onset and the incidence of hemodynamic failure without affecting mortality in a short/long-term [<30 days RR = 0.83, 95% CI (0.47, 1.46);≥30 days RR = 1.04, 95% CI (0.88, 1.22)]. However, tenecteplase was associated with high bleeding risk [<30 days RR = 1.79, 95% CI (1.61, 2.00); ≥30 days RR = 1.28, 95% CI (0.62, 2.64)].

**Conclusions:**

Tenecteplase may represent a promising candidate for patients with high risk PE. However, tenecteplase is not recommended for patients with intermediate-risk PE because of high bleeding risk. More large-scale studies focused on tenecteplase are still needed for PE patients.

## Highlights

- Our study is the first, largest and most comprehensive meta-analysis of the efficacy and safety of tenecteplase on PE.
- For high-risk PE, tenecteplase may be beneficial in improving 30-day survival rate without increasing hemorrhage incidents.
- For intermediate-risk PE, tenecteplase could reduce the risk of hemodynamic decompensation, but was associated with high bleeding risk. Catheter-directed thrombolysis with low-dose teneteplase may be beneficial.

## Background

Pulmonary embolism (PE) is a cardiovascular disease of major global burden after acute coronary syndrome and stroke (1). The estimated incidence of PE ranges from 39 to 115 per 100 000 population worldwide and PE is a major cause of death from cardiovascular disease (2–4). According to 2019 guideline of the European Society of Cardiology/the European Respiratory Society (2019 ESC/ERS), risk stratification of patients with acute PE is classified as high, intermediate and low risk (5). As the guideline recommends, real-world studies also emphasize the management of PE to be guided by risk stratification (3). Reduction of right ventricular dysfunction (RVD) and recurrent PE by reperfusion to reconstruct blood flow and stabilize hemodynamics are major goals in the treatment of acute PE, especially in intermediate-high/high risk PE (6). Conventional treatment of PE mainly refers to anticoagulation therapy including parenteral anticoagulation, such as low-molecular weight heparin (LMWH) or unfractionated heparin (UFH), and direct oral anticoagulants (DOACs). It has been reported that compared with anticoagulation, thrombolytic therapy may improve right-ventricular wall motion at 24 h from baseline (7). Evidence showed that for patients with high-risk PE, thrombolytic therapy reduced mortality and recurrence of PE significantly (8), while its application in intermediate-risk PE was still controversial (9).

Thrombolytic treatment has been shown to increase the risk of hemorrhage. In Pulmonary Embolism Thrombolysis (PEITHO) trial, they found fibrinolytic therapy was associated with a 2.0% rate of hemorrhagic stroke and a 6.3% rate of major extracranial hemorrhage for patients with intermediate-risk PE (10).

Tenecteplase, a genetically modified variant of alteplase, has been proved its potential in the treatment of stroke and cardiovascular disease (11–14). Compared with existing thrombolytic agents, such as alteplase (2-h infusions), tenecteplase can be administered in single intravenous bolus over 5 s due to its long half-life. In addition, unlike streptokinase, an antigenic thrombolytic agent, tenecteplase is less likely to cause allergic reactions (15–18).

Many studies have been conducted on PE patients with tenecteplase, but results were inconsistent (10, 19–23). Therefore, we aimed to summarize the efficacy and safety data of tenecteplase compared with anticoagulant therapy in patients with PE.

## Methods

This meta-analysis was performed according to the guidelines of the Preferred Reporting Items for Systematic reviews and Meta-Analyses (PRISMA) statement.

### Search Strategy

We searched the electronic bibliographic databases systematically, including EMBASE (Excerpta Medica database), PubMed (US National Library of Medicine National Institutes of Health) and Web of Science (including Science Citation Index and Social Sciences Citation Index). Boolean expressions were used when drafting search strategies specifically for each database search engine. The expression included the items (Supplementary Table 2 shows the full electronic search strategy for all database): (Tenecteplase OR TNK-tPA OR TNK-tissue plasminogen activator OR Metalyse OR TNKase) AND (Pulmonary Infarction OR pulmonary embolism OR PE OR venous thromboembolism OR venous thrombosis OR Pulmonary Veno-occlusive Disease) AND (Case Control OR clinical trial). We completed the literature search on May 31, 2021. The major articles were also checked for missing hits. The search retrieved 160 references for further filtration.

### Selection Criteria

The retained articles for the meta-analysis should meet the following inclusion criteria simultaneously: (I) aimed to assess the efficacy and safety of tenecteplase on PE; (II) contained information including sample size, patients'outcomes (efficacy and safety outcomes), as well as the necessary statistical measures; (III) was written by English; (IV) Age>17 years; (V) randomized, controlled trials (RCTs) or cohort studies. The quality of RCTs was assessed by the Jadad scale and cohort studies were assessed by the Newcastle-Ottawa Scale (NOS) score. Studies with Jadad scale or NOS score <4 were excluded. Besides, review or meta-analysis, basic medical research, guideline/ case report, and articles with no available data were also excluded. The eligible articles were accessed independently by two members of the present study, according to the criteria above, and a third party was involved if there was any disagreement.

### Data Extraction

From each eligible article, the following information was extracted: surname of the first author, year of publication, country, study design, demographic characteristics of the study population including age, sex and weight, treatment protocols and tenecteplase doses, duration of follow-up, sample size, vital signs such as heart rate and systolic blood pressure, improvement of clinical symptom or RVD index, incidence of recurrent PE, patients who needed to upgrade treatment and all-cause mortality (<30 days and ≥30 days), incidence of hemorrhage (<30 days and ≥30 days) and chronic thromboembolic pulmonary hypertension (CTEPH). Of note, patients requiring upgraded treatment were defined as those with circulatory or respiratory failure but excluding those who died. Discrepancies were solved by discussion among the authors of this study.

### Statistical Analysis

The pooled RRs and 95% CI for death and major bleeding were calculated using a random-effect model. Analyses were conducted according to duration of follow-up (<30 days or ≥30 days). The heterogeneity between studies was assessed by the inconsistency index *I*^2^ statistic (ranging from 0 to 100%) on the basis of the Cochrane *Q* test. Heterogeneity is considered to be low between the studies if *I*^2^ ranged from 0 to 25%, moderate from 25 to 75% and high from 75 to 100%. All the statistical analyses above were performed using STATA software (StataCorp, Texas, USA, version 14.0 for Windows).

## Results

### Qualified Studies

**Figure 1:**
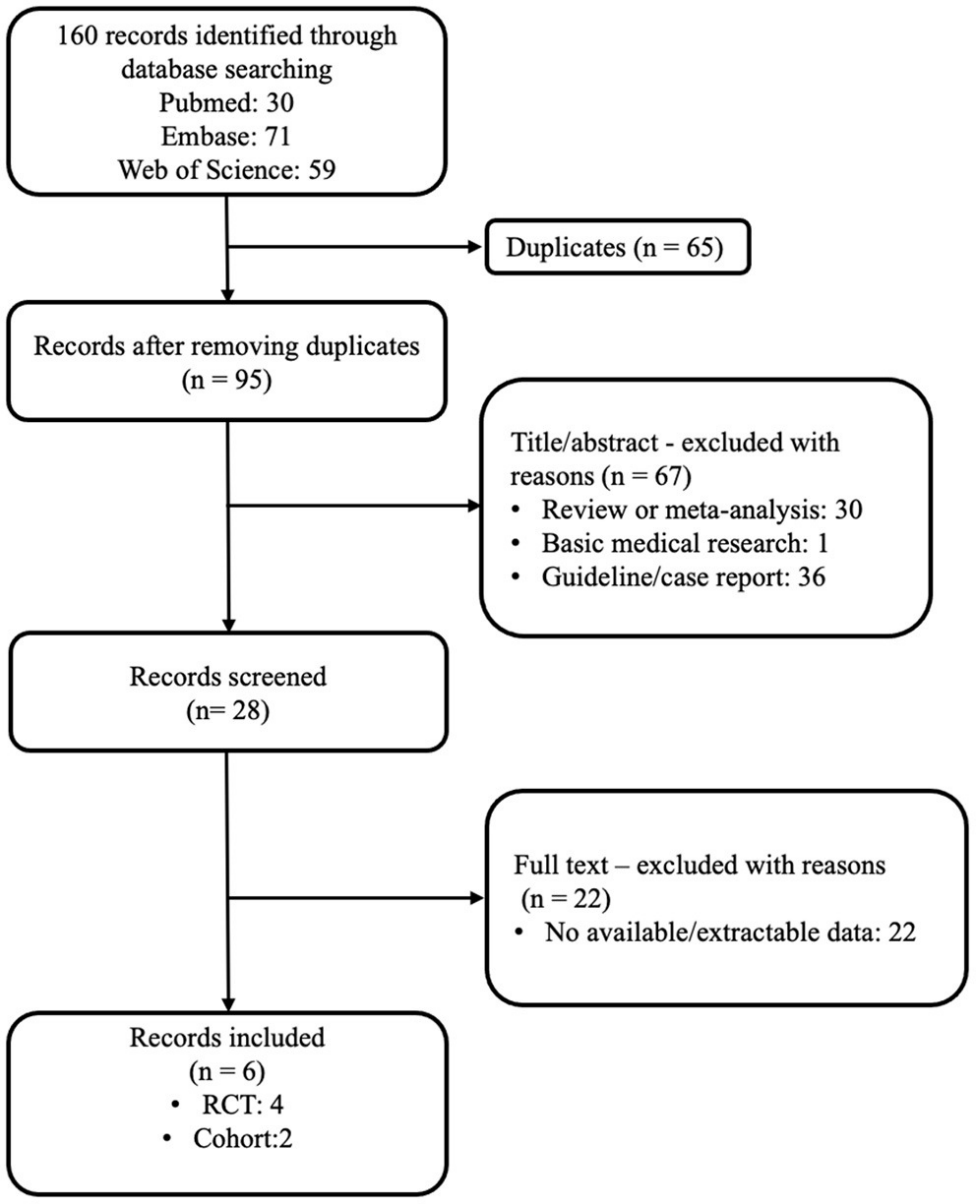
Flow chart of article selection in this study.

According to our search strategy, 160 articles were obtained by the primary literature retrieval from databases. After screening title, abstract and full-text, six articles were identified according to the inclusion criteria. The selection process was visually shown in detail in a flow diagram (Figure 1). The baseline characteristics of the six qualified studies are shown in Table 1. All studies were PE-related. The years of publication ranged from 2010 to 2019, with a total of 2201 patients. Four studies were RCTs (10, 19, 21, 22), and two studies were retrospective/prospective cohort studies. (20, 23) Among the six studies, only one study focused on high-risk PE, four RCT studies included patients with intermediate risk, and one study included both intermediate-risk and high-risk PE patients. (10, 19, 21–23) Doses of tenecteplase ranged from 30 to 50 mg (0.5 mg/kg), with a 5 mg step-up for every 10 kg increase from 60 to 90 kg. All studies scored ≥4 by the Jadad scale or NOS score, where appropriate.

**Table 1:** Characteristics of the studies included in this analysis.

**References**	**Country**	**Study type**	**Risk level**	**Treatment**	**Control**	**Blind**	**Dose**	**Sample size (T/C)**	**Male (T/C)**	**Follow-up days**	**NOS /Jaded score**
Javaudin et al. (20)	France	Retrospective cohort study	High	Fibrinolysis	No fibrinolysis therapy	Assessor-blinded	①	58/188	30/87	30	8
Becattini et al. (19)	Italy	RCT	Intermediate	Tenecteplase	Heparin	Double-blinded	①	28/30	13/10	30	4
Kline et al. (21)	US	RCT	Intermediate	Tenecteplase	Heparin	Double-blinded	①	40/43	20/29	5/90	7
Meyer et al. (10)	Europe	RCT	Intermediate	Tenecteplase	LMWH	Double-blinded	①	506/499	242/231	7/30	7
Konstantinides et al. (22)	Europe	RCT	Intermediate	Tenecteplase	Heparin	Double-blinded	①	359/350	169/159	30/720	8
Patra et al. (23)	India	Prospective cohort study	High/intermediate^*^	Tenecteplase	Streptokinase	NA	①	25/75	15/50	1/7	7

*Tenecteplase: a median dose of 45 mg (minimum, 35;maximum, 50); Alteplase: median dose, 50 mg (minimum, 50; maximum, 80); streptokinase: dose unknown); T, treatment group; C, control group; LMWH, low molecular weight heparin; RCT, randomized controlled trial; NA, not available; NOS, Newcastle-Ottawa Scale*.

* *Patra et al. classified patients as high-risk and intermediate-risk with 57 and 43 individuals, respectively*.

### Tenecteplase May Be Beneficial for Survival Rate Without Increasing Hemorrhage Events in High Risk PE

A study focused on patients with PE and out-of-hospital cardiac arrest from France included 246 patients with PE (Table 1) (20). They followed patients up to 30 days and found that patients receiving thrombolysis therapy during cardiopulmonary resuscitation had a higher 30-day survival rate (16 vs 6%, *p* = 0.005) without association to thrombolytic agents. Tenecteplase was the most used agent in the study (74%). Among 9 survivors in thrombolysis group, tenecteplase was administered to five patients and alteplase was administered to four patients. Moreover, thrombolysis therapy did not increase the mortality rate due to hemorrhage (6 vs 5%; *P* = 0.73).

### Tenecteplase Could Improve the Right Heart Function but Increase the Major Bleeding Risk for Intermediate-Risk PE Patients

Among the five qualified studies for intermediate-risk PE, four studies were RCTs and one was a cohort study (10, 19, 21–23). Tenecteplase was used in all studies, combined with heparin in four studies and with LMWH in one study. Table 1 shows the main information of the five studies. Patients were followed up in all studies, with duration of follow-up ranging from 5 days to 720 days. Meyer et al. (10) collected data on the largest number of patients of 1,005, of whom 506 received tenecteplase. The study of Patra et al. (23) was the only cohort study including both high- and intermediate-risk PE patients. Table 2 shows the baseline information of the patients included. Kline et al. (21) and Patra et al. (23) reported the pulmonary arterial systolic pressure (PASP).

**Table 2:** Baseline patient information of studies involved.

**Risk level**	**References**	**Weight, kg (T/C)**	**APE / DVT history, % (T/C)**	**Heart rate, beats per min (T/C)**	**SBP, mmHg (T/C)**	**RVD, % (T/C)**	**PASP, mmHg (T/C)**
High	Javaudin et al. (20)	NA	NA	NA	NA	NA	NA
Intermediate	Becattini et al. (19)	79.0/79.8	NA	90.3/102.0	131.0/129.7	100/100	NA
Intermediate	Kline et al. (21)	NA	15/21	NA	NA	100/100	58/55
Intermediate	Meyer et al. (10)	82.5/82.6	25/30	94.5/92.3	130.8/131.3	100/100	NA
Intermediate	Konstantinides et al. (22)	82.6/81.0	23/27	94.9/91.5	130.6/132.3	100/100	NA
High/intermediate^*^	Patra et al. (23)	NA	80/50	104.0/120.0	108.0/98.0	100/100	58/63

*APE, acute pulmonary embolism; DVT, deep vein thrombosis; SBP, systolic blood pressure; RVD, right ventricular dysfunction; PASP, pulmonary arterial systolic pressure; NA, not available; T, tenecteplase group; C, control group*.

* *Patra et al. classified patients as high-risk and intermediate-risk with 57 and 43 individuals, respectively*.

Table 3 contains information on the clinical events and prognosis of studies involved. In terms of the major efficacy outcome, Meyer et al. (10) concluded that tenecteplase reduced hemodynamic decompensation based on the largest dataset from intermediate-risk PE patients [upgrade therapy: 8(1.6%) in tenecteplase group vs 25(5.0%) in placebo group, p = 0.002)]. Similarly, different studies verified that tenecteplase could improve right ventricle function for patients with intermediate-risk PE. Some studies found that RVD index and PASP at 24 h/7-day reduced to a greater extent in the tenecteplase group than the control group. (19, 23) Moreover, they found lower mean duration of intensive care unit (ICU) stay in the tenecteplase group (*p* = 0.04) (23). In addition to objective indicators such as RVD index, we also collected relatively subjective indicators such as persistence of clinical symptoms. Kline et al. (22) found that 12 (27.9%) patients remained clinically symptomatic in the control group at 90-day follow-up compared to only 4(10.0%) patients in the tenecteplase group (*p* = 0.039). With regard to long-term prognosis, Konstantinides et al. (22), the study with the longest follow-up duration (720 days), found that tenecteplase use was not correlated with persistent clinical symptoms[63(36.0%) vs 55 (30.1%), p = 0.23)], RVD index improvement[81(56.3%) vs 94(64.4%), *p* = 0.20)] or CTEPH morbidity[4(2.1%) vs 6(3.2%), p = 0.79)]. Furthermore, currently available data did not demonstrate a clear association between tenecteplase and recurrent PE events.

**Table 3:** Clinical events and prognosis of studies involved.

**References**	**Improvement of RVD index (n, T/C)**	**Persistence of clinical symptom during short-term follow-up (<3 months) [n (%), T/C]**	**Persistence of clinical symptom during long-term follow-up (≥3 months) [n (%), T/C]**	**Recurrent APE [n (%), T/C]**	Upgrade therapy [n (%), T/C]^*^	**All-cause mortality during short-term follow-up (<30 days) [n (%), T/C]**	**All-cause mortality during long-term follow-up (≥30 days) [n (%), T/C]**	**Major bleeding [n (%), T/C]**	**Minor bleeding [n (%), T/C]**	**CTEPH [n (%), T/C]**
Javaudin et al. (20)	NA	NA	NA	NA	9 (15.5)/11 (5.9)	NA	49 (84.5)/176 (93.6)	3 (5.2)/9 (4.8)	NA	NA
Becattini et al. (19)	0.31/0.10	NA	NA	1 (3.3)/1 (3.6)	0 (0)/1 (3.3)	NA	0 (0)/1 (3.3)	2 (7.1)/1 (3.3)	13 (46.4)/1 (3.3)	NA
Kline et al. (21)	NA	NA	4 (10.0)/12 (27.9)	0 (0)/3 (7.0)	0 (0)/2 (4.7)	1 (2.5)/1 (2.3)	0 (0)/0 (0)	1 (2.5)/0 (0)	NA	NA
Meyer et al. (10)	NA	NA	NA	1 (0.2)/5 (1.0)	8 (1.6)/25 (5.0)	6 (1.2)/9 (1.8)	12 (2.4)/16 (3.2)	90 (17.8)/18 (3.6)	165 (32.6)/43 (8.6)	NA
Konstantinides et al. (22)	81 (56.3%)/94 (64.4%)	NA	63 (36.0);55 (30.1)	0 (0)/2 (0.6)	1 (0.3)/1 (0.3)	NA	73 (20.3)/63 (18.0)	1 (0.3)/1 (0.3)	NA	4 (2.1); 6 (3.2)
Patra et al. (23)	23 (92.0%)/66 (88.0%)	NA	NA	NA	5 (20.0)/19 (25.3)	2 (8.0)/6 (8.0)	NA	0 (0)/1 (1.3)	3 (12.0)/13 (17.3)	NA

*RVD, right ventricular dysfunction; APE, acute pulmonary embolism; CTEPH, chronic thromboembolic pulmonary hypertension; NA, not available; T, Tenecteplase group; C, Control group*.

* *patients requiring upgraded therapy are defined as those with circulatory or respiratory failure but excluding those who died*.

**Figure 2:**
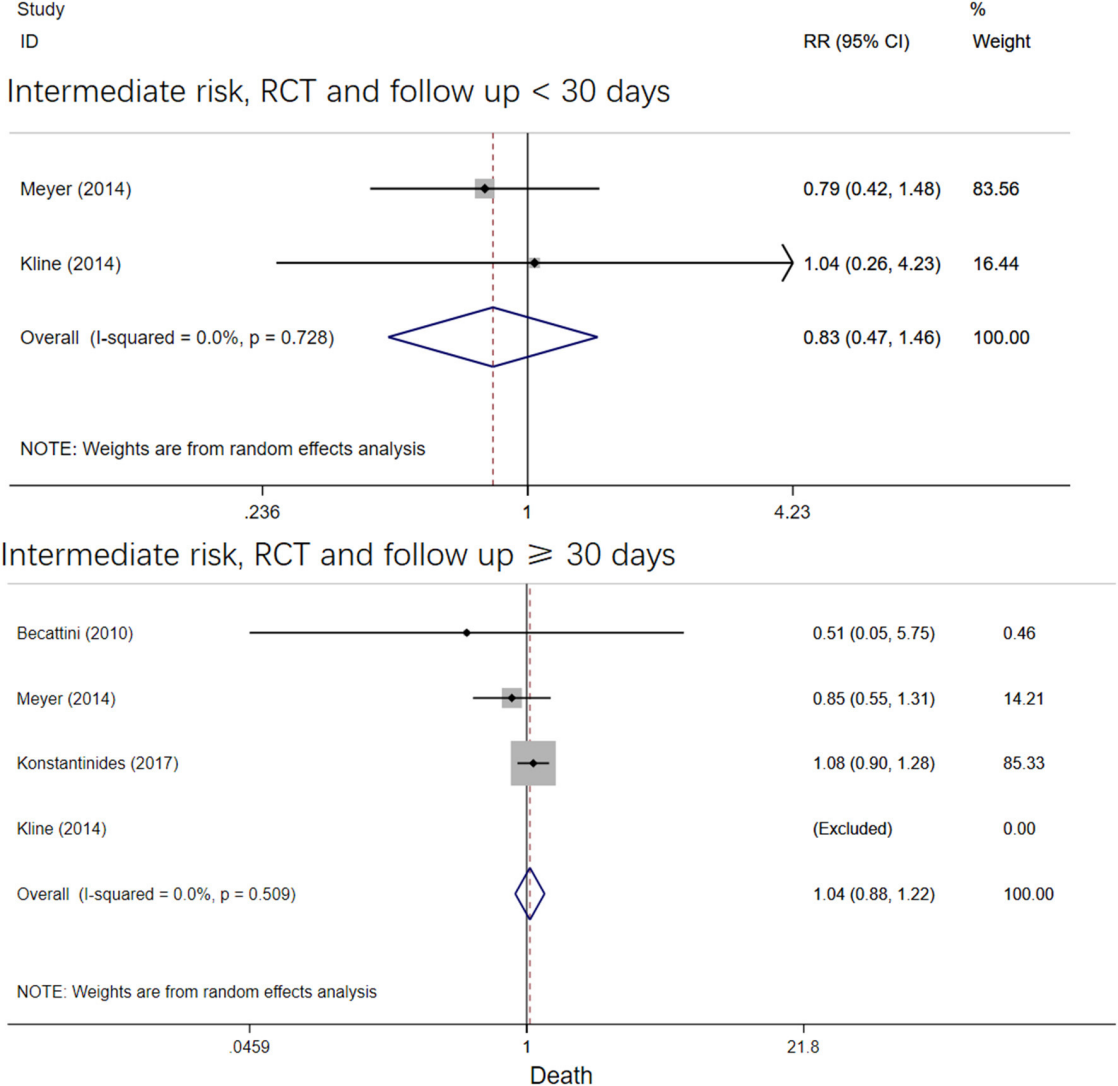
Forest plots of tenecteplase vs. anticoagulation treatment grouped by all-cause mortality and duration of follow-up (<30 days or≥30 days) for patients with intermediate-risk PE.

**Figure 3:**
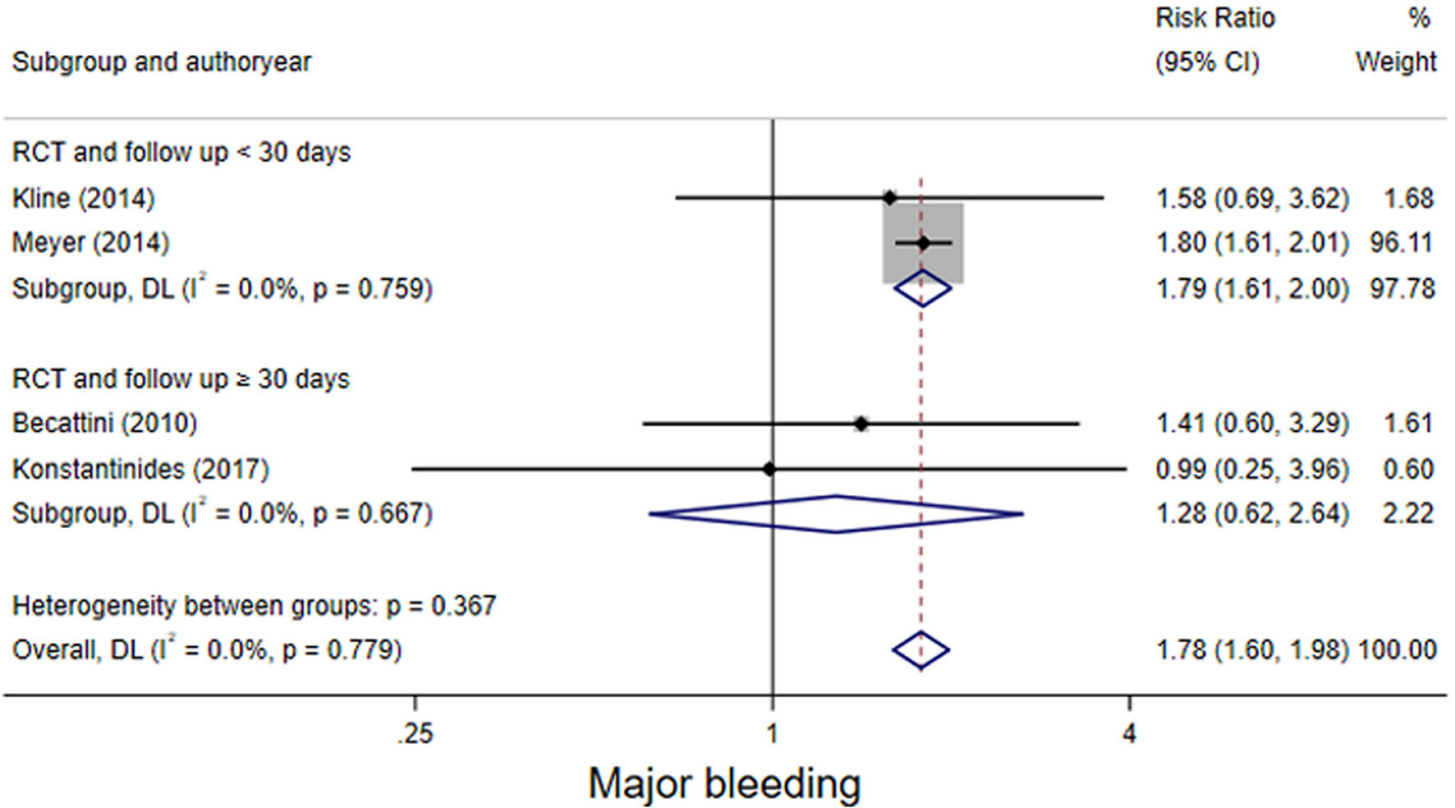
Forest plots of tenecteplase vs. anticoagulation treatment grouped by hemorrhage rates (<30 days or≥30 days) for patients with intermediate-risk PE.

**Figure 4:**
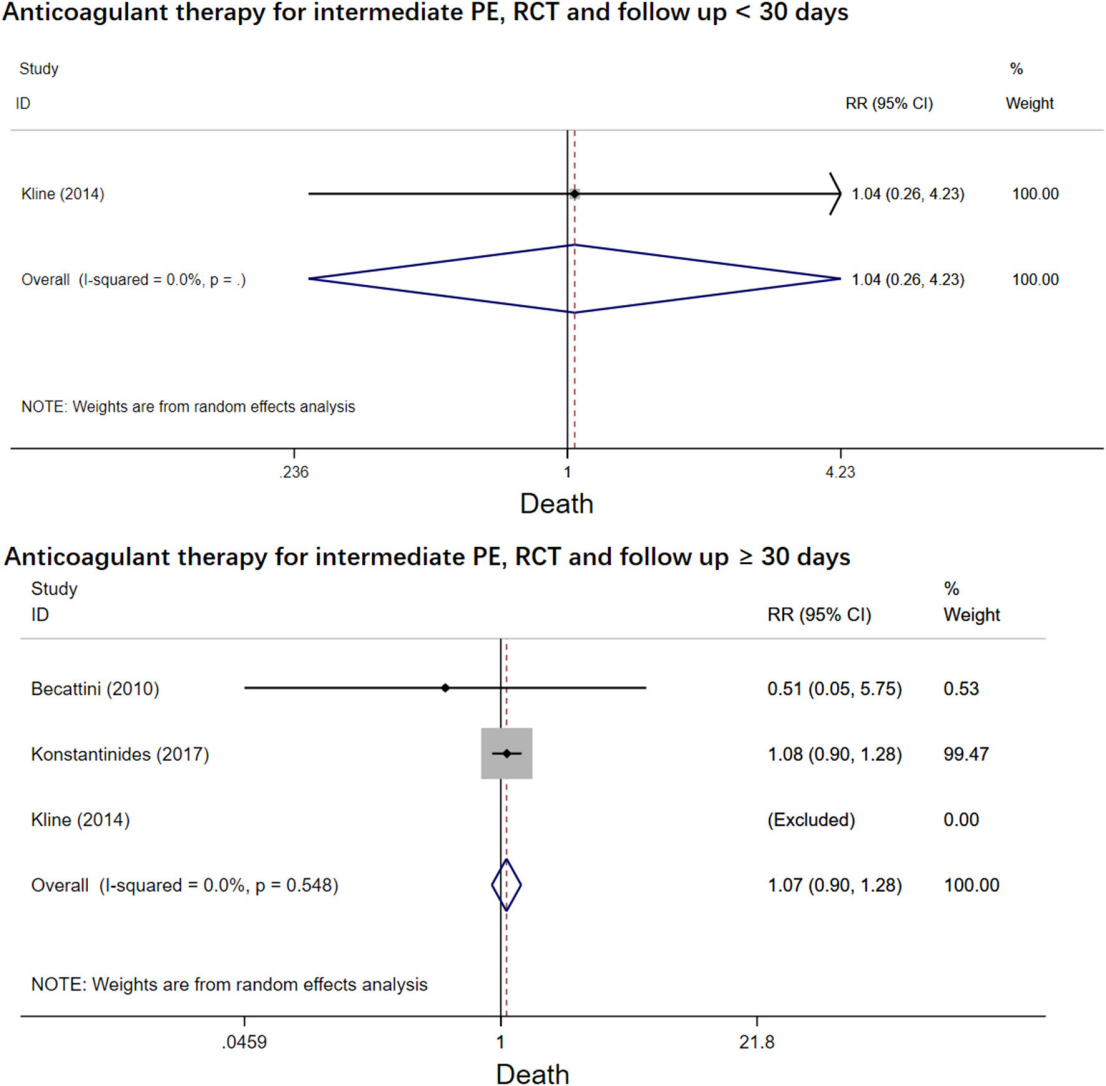
Forest plots of tenecteplase vs. anticoagulation treatment grouped by all-cause mortality and duration of follow-up (<30 days or≥30 days) for patients with intermediate-risk PE (excluding Meyer et al. study).

**Figure 5:**
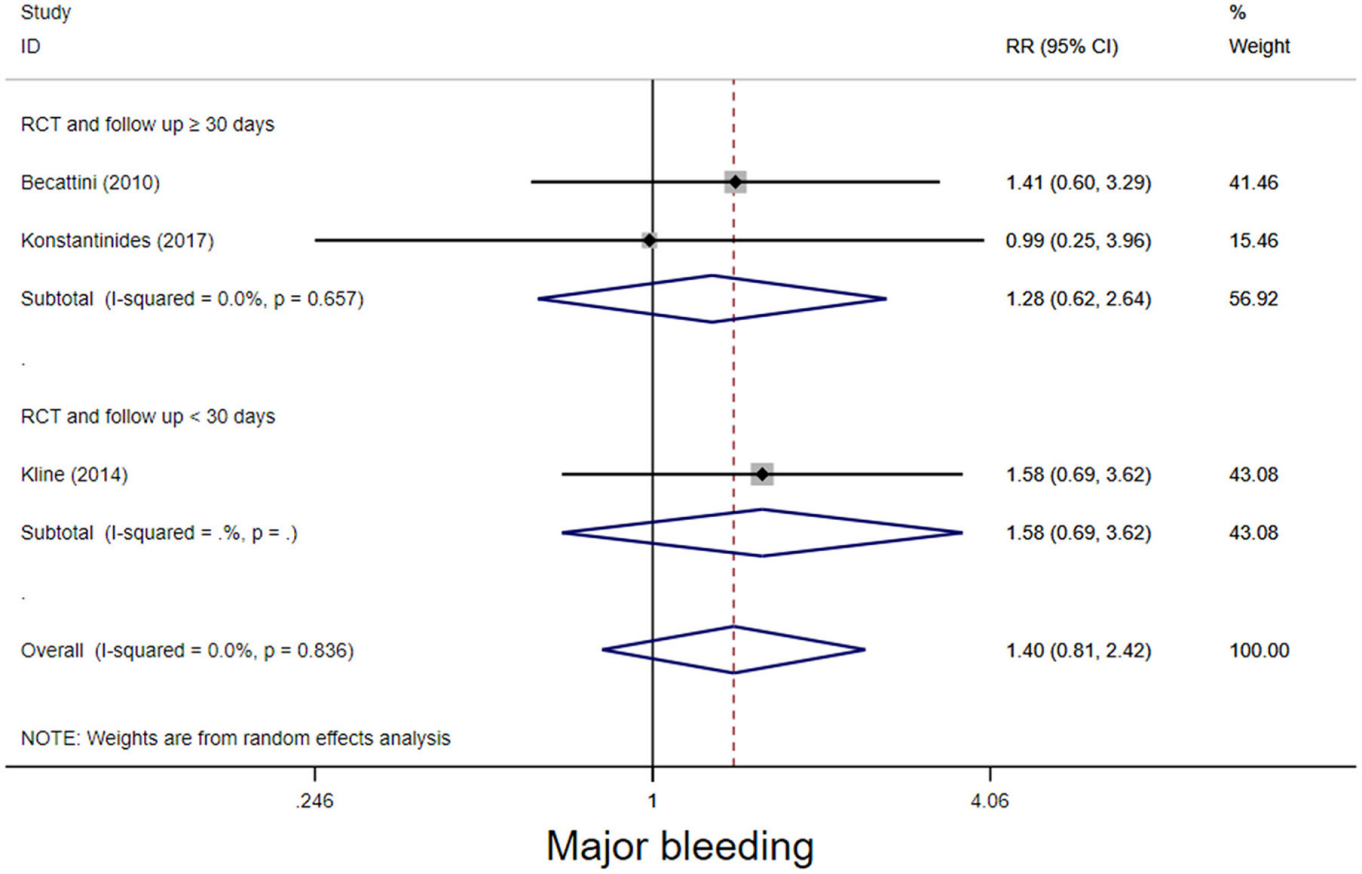
Forest plots of tenecteplase vs. anticoagulation treatment grouped by hemorrhage rates (<30 days or≥30 days) for patients with intermediate-risk PE. (excluding Meyer et al. study).

The four RCT studies had low heterogeneity (*I*^2^ = 0.0%), all of which suggested that tenecteplase did not affect short- and long-term mortality in PE patients (10, 19, 21, 22). Compared with coagulation treatment in patients with intermediate-risk PE, the pooled RRs of tenecteplase in all-cause mortality were 0.83 [95% CI (0.47, 1.46)] with a follow-up of <30 days (Figure 2) and 1.04 [95% CI (0.88, 1.22)] with a follow-up of ≥30 days, respectively (Figure 2). Additionally, the pooled RRs of tenecteplase in major bleeding were 1.79[95% CI (1.61, 2.00)] with a follow-up of <30 days (Figure 3) and 1.28 [95% CI (0.62, 2.64)] with a follow-up of ≥30 days, respectively (Figure 3). However, Meyer et al. (10) is the largest trial and may have some influence on overall analysis. We also performed overall mortality and bleeding rates excluding the Meyer study. The all-cause mortality rate RRs were 1.04 [95% CI (0.26, 4.23)] with a follow-up of <30 days (Figure 4) and 1.07 [95% CI (0.90, 1.28)] with a follow-up of ≥30 days, respectively (Figure 4). The major bleeding rates RRs were 1.40 [95% CI (0.81, 2.42)] with a follow-up of <30 days (Figure 5) and 1.28 [95% CI (0.62, 2.64)] with a follow-up of ≥30 days, respectively (Figure 5).

## Discussion

To our knowledge, the present study is the first, largest and most comprehensive meta-analysis of the efficacy and safety of tenecteplase in PE patients, summarizing multiple RCT and cohort studies. There are several key points from this meta-analysis and systematic review. First, for patients with high-risk PE, tenecteplase could improve patient survival over 30 days without increasing major bleeding rates. Second, for patients with intermediate-risk PE, tenecteplase could prevent the disease progression and improve the clinical symptoms rapidly, decreasing the length of ICU stay and cost. Furthermore, tenecteplase has some unique advantages such as high fibrin specificity and convenient usage. However, tenecteplase could increase the major bleeding risk in the short term as could other thrombolytic agents. In summary, we believe tenecteplase is a promising candidate for patients with high risk PE. Further studies related to tenecteplase are quite necessary, especially for patients with high risk PE.

As a third-generation thrombolytic agent, tenecteplase has been widely studied in thrombotic diseases due to its unique advantages. We summarized the advantages and disadvantages of the different thrombolytic agents in Supplementary Table 1. (24) In comparison, tenecteplase has more advantages. First of all, tenecteplase demonstrates the greatest fibrin specificity, decreasing the risk of major bleeding. Secondly, the clearance of tenecteplase is approximately eight-fold slower than alteplase. In contrast, alteplase requires a continuous intravenous infusion for 2 h while tenecteplase is administered in 5–10 min by a single bolus (11, 25). Moreover, tenecteplase has been under research in other thrombotic studies such as acute myocardial infarction (AMI) and acute ischemic stroke (AIS) (Tables 4, 5). In 2000, tenecteplase has been approved to treat AMI by the Food and Drug Administration (FDA), as it reduces the risk of major bleeding with the similar efficacy compared to alteplase (12). Although tenecteplase has not yet received FDA approval for AIS, a meta-analysis found tenecteplase was noninferior to alteplase and improved the neurologic function in the early stage (26). Also, tenecteplase may reduce the delay in endovascular thrombectomy and may be more suitable for large vessel occlusions because of convenient usage (26). These studies provide a basis and demonstrate the potential for tenecteplase in PE studies.

**Table 4:** Tenecteplase therapy in patients with thrombotic diseases.

	**Time of FDA approval**	**Advantages**	**Disadvantages**
AMI	2000	➢ Similar efficacy with alteplase in reperfusion therapy. ➢ Reducing the risk of major bleeding	➢ 30-day mortality was similar in patients receiving alteplase
AIS	Not approved	➢ Higher rates of both recanalization and early neurological improvement. ➢ Not increasing intracerebral bleeding or mortality. ➢Noninferior to alteplase in treatment	➢ All-at-once administration and longer serum half-life may allow hemostasis to return more quickly
APE	Not approved	➢ Similar efficacy and safety as streptokinase, heparin and alteplase. ➢ May decrease duration of stay in the ICU. ➢ May have higher rates of improvement in clinical symptoms and SaO_2_	➢ Increases the risk of major bleeding over anticoagulation for intermediate-risk APE

*AMI, acute myocardial infarction; AIS, acute ischemic stroke; APE, acute pulmonary embolism; FDA, Food and Drug Administration; ICU, intensive care unit; SaO_2_, blood oxygen saturation*.

**Table 5:** The meta-analyses of tenecteplase in patients with thrombotic diseases.

**Disease type**	**References**	**Number of studies included**	**Main findings**
AMI	Guillermin et al. (12)	4	Tenecteplase reduces the risk of major bleeding with the similar efficacy as alteplase in the treatment of AMI.
AIS	Burgos et al. (26)	5	Tenecteplase is noninferior to alteplase in the treatment of AIS.
APE	Our study	6	Tenecteplase is recommended for patients with intermediate/high-risk APE.

*AMI, acute myocardial infarction; AIS, acute ischemic stroke; APE, acute pulmonary embolism*.

High-risk PE, defined by sustained hypotension or cardiogenic shock, is associated with a 24-h short-term mortality rate>20%. Despite limited study, high-risk PE is a clear indication for thrombolytic therapy according to guidelines including the 2019 ESC/ERS and American College of Chest Physicians (5, 27). Pooled data from several systematic reviews and meta-analyses support an increased survival benefit with thrombolytic therapy when used in patients with high-risk PE (28). A large prospective cohort study concluded that thrombolysis during cardiopulmonary resuscitation was associated with higher 30-day survival rate without increasing the rate of hemorrhage in high-risk PE patients, whether the thrombolytic agent was tenecteplase or alteplase (20). Therefore, the tenecteplase may benefit patients with high-risk PE in efficacy and safety aspects and need further studies to verify the point in the future.

The current evidence included studies that mainly focused on intermediate-risk PE group. The PEITHO study, the largest randomized, placebo-controlled trial of fibrinolysis for intermediate-risk PE to date, found tenecteplase was associated with reduced hemodynamic decompensation at 7 days (1.6 vs. 5.0%, *p* = 0.002). From Table 3, we concluded that tenecteplase could reduce the risk of hemodynamic failure for these normotensive PE patients (10), which indicated that tenecteplase may prevent the further disease progression. Additionally, tenecteplase was better than UFH at reducing RVD in the early stage (19) but did not affect short/long-term mortality. Compared with streptokinase, studies have found that tenecteplase could improve the clinical symptoms rapidly and enable patients to obtain better self-assessment of overall health function, especially for those with comorbid conditions such as recurrent venous thromboembolism or heart failure, which was also verified by Stewart et al. and Agrawal et al. (21, 23, 29–31). Similarly, an observational study found that tenecteplase could reduce heart rate, increase the systolic blood pressure and oxygen saturation (29). Furthermore,tenecteplase could decrease the dependency for ICU and the length of stay, therefore, the application of tenecteplase may reduce the cost of therapy (21, 23).

However, current results on the risk of bleeding with tenecteplase are controversial. Clinicians are cautious about thrombolytic therapy mainly because of the concerns of bleeding. It is noted that the risk of bleeding generally remains elevated for a period of 12–24 h after thrombolytic infusion (28). Becattini et al. (19) found that tenecteplase did not increase excessive major bleeding rates. While data from PEITHO trial (10) believed that teneteplase increased the risk of major bleeding, including intracranial hemorrhage, within 7 days [90(17.8%) vs. 18(3.6%), *p* < 0.001)]. Pooled data also showed that the frequency of major bleeding in patients treated with systemic fibrinolytic therapy is 0–33% and the incidence of intracranial hemorrhage is 0–7.4% (32). According to our meta-analysis, tenecteplase was associated with higher bleeding risk in 7 days for intermediate-risk PE patients and did not affect long-term bleeding events. As the guideline indicated, we believed tenecteplase, similar to other thrombolytic agents, increased the risk of bleeding for aged patients who have more comorbidities. However, some retrospective studies showed that tenecteplase did not increase, but reduced the hemorrhagic rates (23, 31). We speculated that the differences in some studies was associated with drug doses and its administration. The current doses of tenecteplase were 0.5 mg/kg in most studies involved, with a 5 mg step-up for every 10 kg increase from 60 to 90 kg; however, the 0.25 mg/kg dose of tenecteplase was found to be associated with early neurological improvement and reduced tendency of intracranial hemorrhage compared to other thrombolytic agents in the treatment of stroke (33). Our previous study on thrombolysis also showed that half-dose thrombolysis reduced the risk of bleeding with similar efficacy (34) Moreover, applying catheter-directed thrombolysis with tenecteplase to treat PE patients with RVD appeared to improve right ventricle function without increasing bleeding risk (35). Recently, the HI-PEITHO study launched and started enrollment, which aims to assess whether ultrasound-facilitated, catheter-directed thrombolysis and standard anticoagulation are associated with adverse outcomes for patients with intermediate-high risk PE (36). Therefore, catheter-guided administration of low-dose teneteplase may benefit patients with intermediate-risk PE. In conclude, we do not recommend tenecteplase for intermediate-risk PE patients based on current evidence, further studies would be necessary to validate the efficacy and safety of tenecteplase at a lower dose or the different methods of administration.

High-risk PE patients may be suitable for tenecteplase, however, for patients with intermediate-risk PE, it was not appropriate to apply tenecteplase with the same dose or regimen as with high-risk PE patients. Studies have reported that normotensive PE patients with elevated troponin and BNP, or lactate≥ 2 mmol/L were at a higher risk of the adverse outcomes, and indicated a potential need for more aggressive systemic thrombolytic treatment instead of anticoagulants alone in these patients. In this way, these patients should be closely monitored, and teneteplase could be beneficial when hemodynamic instability occurs.

We acknowledge some limitations of our analysis. First, a publication bias is possible, however, as the number of studies included was limited, no filled funnel plot for publication bias or Egger's test was generated or performed. Second, the sample sizes of some subgroups were too small to assess heterogeneity between studies and draw an accurate conclusion. Third, the PE risk level was not available for all involved studies, which may lead to misclassification. Also, only one study focused on the treatment of tenecteplase in high-risk PE patients may cause bias.

## Conclusion

In conclusion, our study indicated that tenecteplase would be suitable for high-risk PE patients because it could be beneficial for 30-day survival rate without increasing hemorrhagic incidents. However, tenecteplase is not recommended for patients with intermediate-risk PE because of high bleeding risk. More large-scale studies focused on catheter-directed thrombolysis involving intermediate-high/high risk PE are needed to validate the efficacy and safety of tenecteplase on short/long-term outcomes.

## Data Availability Statement

The original contributions presented in the study are included in the article/Supplementary Material, further inquiries can be directed to the corresponding author.

## Author Contributions

ZZhai and CW have full access to all the data in the study and take responsibility for the content of the manuscript. ZZhan conceived and designed the study. ZZhan and LX integrated data, analyzed the data, and wrote the manuscript. GF provided methodological support. PY participated in editing of the manuscript. SZ, YZ, XT, QG, and WX contributed to the interpretation of the data and clinical inputs. All authors were involved in the revision of the manuscript for important intellectual content and approved the final version.

## Funding

This study is funded by the by CAMS Innovation Fund for Medical Sciences(CIFMS) (2021-I2M-1-061) and The National Key Research and Development Program of China (No. 2016YFC0905600).

## Conflict of Interest

The authors declare that the research was conducted in the absence of any commercial or financial relationships that could be construed as a potential conflict of interest.

## Publisher's Note

All claims expressed in this article are solely those of the authors and do not necessarily represent those of their affiliated organizations, or those of the publisher, the editors and the reviewers. Any product that may be evaluated in this article, or claim that may be made by its manufacturer, is not guaranteed or endorsed by the publisher.

## Abbreviations

PE: pulmonary embolism
RRs: risk ratios
PEITHO: Pulmonary Embolism Thrombolysis
CI: confident intervals
RCTs: randomized controlled trials
RVD: right ventricular dysfunction
LMWH: low molecular weight heparin
UFH: unfractionated heparin
DOACs: direct oral anticoagulants
tPA: tissue plasminogen activator
2019 ESC/ERS: 2019 Guideline of the European Society of Cardiology/the European Respiratory Society
PRISMA: preferred reporting items for systematic reviews and meta-analyses
NOS: Newcastle-ottawa scale
CTEPH: chronic thromboembolic pulmonary hypertension
PASP: pulmonary artery systolic pressure
ICU: intensive care unit
AMI: acute myocardial infarction
AIS: acute ischemic stroke
FDA: Food and Drug Administration.
