# Supplementary material for: Tenecteplase in Pulmonary Embolism Patients: A Meta-Analysis and Systematic Review
Source: Front Med (Lausanne). 2022 Mar 31;9:860565. doi: 10.3389/fmed.2022.860565 (PMC9008780; doi:10.3389/fmed.2022.860565)
Supplement: Supplementary file 1 [file Data_Sheet_1.pdf]

Supplement Table 1 Comparison of Tenecteplase with other thrombolytic drugs.

Thrombolytic Drug	Advantages	Disadvantages
Tenecteplase	➢ Third-generation thrombolytic agent<br>➢ Single bolus administration<br>➢ High fibrin specificity<br>➢ Long half-life<br>➢ May protect heath workers against COVID-19	➢ May increase the bleeding risk<br>➢ Limited clinical studies and data<br>➢ No antagonist drugs
Alteplase	➢ Second-generation thrombolytic agents<br>➢ Abundant clinical studies and data<br>➢ Relatively low bleeding risk<br>➢ Stoppable at any time	➢ Administration requires 2h intravenous infusion<br>➢ Short half-life<br>➢ No antagonist drugs
Streptokinase	➢ First-generation thrombolytic agents<br>➢ Low cost and easily accessible	➢ Prone to allergic reactions<br>➢ Relatively high bleeding risk<br>➢ Low fibrin specificity<br>➢ No antagonist drugs
Urokinase	➢ First-generation thrombolytic agents<br>➢ Low cost and easily accessible	➢ Relatively high bleeding risk<br>➢ Low fibrin specificity

	➢ No antagonist drugs

Notes: COVID-19, coronavirus disease 2019.

Supplement Table 2 Full electronic search strategy for all databases.

Database	Full electronic search strategy
PubMed	Tenecteplase[Title/Abstract] OR TNK-tPA [Title/Abstract]OR TNK-tissue plasminogen activator [Title/Abstract]OR Metalyse[Title/Abstract] OR TNKase[Title/Abstract]) AND (Pulmonary Infarction[Title/Abstract] OR PE[Title/Abstract] OR venous thromboembolism[Title/Abstract] OR venous thrombosis[Title/Abstract] OR Pulmonary Veno-Occlusive Disease[Title/Abstract] OR venous thromboembolism[Title/Abstract] OR venous thrombos*[Title/Abstract] OR deep ve* thrombos*[Title/Abstract] OR deep ve* thrombus[Title/Abstract] OR deep ve* thromboembolism[Title/Abstract] OR pulmonary embol*[Title/Abstract] OR pulmonary thromb*[Title/Abstract]) AND (Case Control[Title/Abstract] OR Case-Control[Title/Abstract] OR Case-Referent[Title/Abstract] OR Case Referent[Title/Abstract] OR Case-Compeer[Title/Abstract] OR Case Comparison[Title/Abstract] OR Case-Comparison[Title/Abstract] OR Case Base[Title/Abstract] OR Case-Base[Title/Abstract] OR Retrospective[Title/Abstract] OR clinical trial*[Title/Abstract] OR clinical trial[Publication Type] OR Intervention Study[Title/Abstract] OR Randomized Controlled Trial[Title/Abstract] OR Non-Inferiority Trial[Title/Abstract] OR Noninferiority Trial[Title/Abstract] OR Superiority Trial[Title/Abstract] OR (clinical[Title/Abstract] AND trial[Title/Abstract]) OR random*[Title/Abstract] OR random allocation[Title/Abstract] OR therapeutic use[Title/Abstract] OR Randomized Controlled Trial[Publication Type]
Embase	((Tenecteplase):ab,ti OR (TNK-tPA):ab,ti OR (TNK-tissue plasminogen activator):ab,ti OR (Metalyse):ab,ti OR (TNKase):ab,ti) AND ((Pulmonary Infarction):ab,ti OR (PE):ab,ti OR (venous thromboembolism):ab,ti OR (venous thrombosis):ab,ti OR (Pulmonary Veno-Occlusive Disease):ab,ti OR (venous thromboembolism):ab,ti OR (venous thrombos*):ab,ti OR (deep ve* thrombos*):ab,ti OR (deep ve* thrombus):ab,ti OR (deep ve* thromboembolism):ab,ti OR (pulmonary embol*):ab,ti OR (pulmonary thromb*):ab,ti) AND ((Case Control):ab,ti OR (Case-Control):ab,ti OR (Case-Referent):ab,ti OR (Case Referent):ab,ti OR (Case-Compeer):ab,ti OR (Case Comparison):ab,ti OR (Case-Comparison):ab,ti OR (Case Base):ab,ti OR (Case-Base):ab,ti OR (Retrospective):ab,ti OR (clinical trial*):ab,ti OR (Intervention Study):ab,ti OR (Randomized Controlled Trial) OR (Non-Inferiority Trial):ab,ti OR (Noninferiority Trial):ab,ti OR (Superiority Trial):ab,ti OR ((clinical):ab,ti AND (trial):ab,ti):ab,ti OR (random*):ab,ti OR (random allocation):ab,ti OR (therapeutic use):ab,ti)
Web of Science	TS = (Tenecteplase OR TNK-tPA OR TNK-tissue plasminogen activator OR Metalyse OR TNKase) AND TS = (Pulmonary Infarction OR PE OR venous thromboembolism OR venous thrombosis OR Pulmonary Veno-Occlusive Disease OR venous thromboembolism OR venous thrombos* OR deep ven* thrombos* OR deep ven* thrombus OR deep ven* thromboembolism OR pulmonary embol* OR pulmonary thromb*) AND TS = (Case Control OR Case-Control OR Case-Referent OR Case Referent OR Case-Compeer OR Case Comparison OR Case-Comparison OR Case Base OR Case-Base OR Retrospective OR clinical trial* OR Intervention Study OR Randomized Controlled Trial OR Non-Inferiority Trial OR Noninferiority Trial OR Superiority Trial OR (clinical AND trial) OR random* OR random allocation OR therapeutic use)
